# Autochthony and isotopic niches of benthic fauna at shallow-water hydrothermal vents

**DOI:** 10.1038/s41598-022-09839-w

**Published:** 2022-04-15

**Authors:** Teng-Wei Wang, Danny C. P. Lau, Tin-Yam Chan, Benny K. K. Chan

**Affiliations:** 1 Institute of Marine Biology and Center of Excellence for the Oceans, National Taiwan Ocean University, Chilung, 202301 Taiwan, ROC; 2 Department of Bioscience, National Chiayi University, Chiayi, 60004 Taiwan, ROC; 3 Department of Aquatic Sciences and Assessment, Swedish University of Agricultural Sciences, SE-75007 Uppsala, Sweden; 4 Department of Ecology and Environmental Science, Umeå University, Umeå, Sweden; 5 Biodiversity Research Center, Academia Sinica, Taipei, 11529 Taiwan, ROC

**Keywords:** Ecosystem ecology, Stable isotope analysis

## Abstract

The food webs of shallow-water hydrothermal vents are supported by chemosynthetic and photosynthetic autotrophs. However, the relative importance of these two basal resources for benthic consumers and its changes along the physicochemical gradient caused by vent plumes are unknown. We used stable carbon and nitrogen isotopes (i.e., δ^13^C and δ^15^N) and Bayesian mixing models to quantify the dietary contribution of basal resources to the benthic fauna at the shallow-water vents around Kueishan Island, Taiwan. Our results indicated that the food chains and consumer production at the shallow-water vents were mainly driven by photoautotrophs (total algal contribution: 26–54%) and zooplankton (19–34%) rather than by chemosynthetic production (total contribution: 14–26%). Intraspecific differences in the trophic support and isotopic niche of the benthic consumers along the physicochemical gradient were also evident. For instance, sea anemone *Anthopleura* sp. exhibited the greatest reliance on chemosynthetic bacteria (26%) and photoautotrophs (66%) near the vent openings, but zooplankton was its main diet in regions 150–300 m (32–49%) and 300–700 m (32–78%) away from the vent mouths. The vent-induced physicochemical gradient structures not only the community but also the trophic support and isotopic niche of vent consumers.

## Introduction

Hydrothermal vents are underwater ecosystems that provide substantial support for the diversity and biomass of organisms. More than 500 animal species belonging to 12 phyla have been described in these ecosystems worldwide^1^. Hydrothermal vents are often found in tectonically active regions and can be classified into two major types on the basis of the depth at which they occur: deep-sea (> 200 m) and shallow-water vents (10–200 m)^2,3^. The first deep-sea hydrothermal vent ecosystem was discovered in the Galapagos Rift in 1976^4^. Deep-sea vents are predominantly driven by chemosynthesis because of the limited light and photosynthetic autotroph production, and the large supply of CO_2_, CH_4_, H_2_S, and other compounds that can be used as energy sources^2^. The food webs at deep-sea vents are typically supported by chemolithotrophic bacteria and by the dominant holobiont (symbiotic chemosynthetic bacteria and their host, including *Bathymodiolus* mussels (Mytilidae)), which are abundant in these ecosystems^1,4–7^.

Unlike deep-sea vents, shallow-water vents are usually located closer to the coast and are found in many regions worldwide, such as Papua New Guinea, the Mediterranean Sea, the Kuril Islands, Baja California, Japan, and Taiwan^3^. They were discovered as early as the deep-sea vents^1,3^, but most ecological studies of vent systems have been directed toward deep-sea vents, and few have focused on shallow-water vents^1–3^. Shallow-water vents are commonly located at the photic zone; thus, energy is supplied by both chemosynthesis and photosynthesis to support the vent food webs^3,8–11^. Shallow-water vents are known to harbor some endemic fauna, such as polychaetes *Haplosyllis cratericola* (Buzhinskaja, 1990) (Syllidae) and *Lamellibrachia satsuma* Miura, 1997 (Siboglinidae), sea spider *Pycnogonum kussakini* Turpaeva, 2000 (Pycnogonidae), and crabs *Xenograpsus* spp. (Xenograpsidae)^3,9^. The assemblages of shallow-water vents are, therefore, different from those of other coastal ecosystems and deep-sea vents^9^. Most studies on shallow-water vents have focused on taxonomic composition, biodiversity, and species responses to environmental variation^10,12,13^. Previous studies have indicated that the production of shallow-water vent fauna relies on chemosynthetic^14–16^ and photosynthetic autotrophs^17,18^. However, few studies have performed a quantitative analysis of chemosynthetic and photosynthetic trophic support for vent food webs and investigated how their role changes along the spatial and physicochemical gradients from vent mouths to more distant waters.

**Figure 1:**
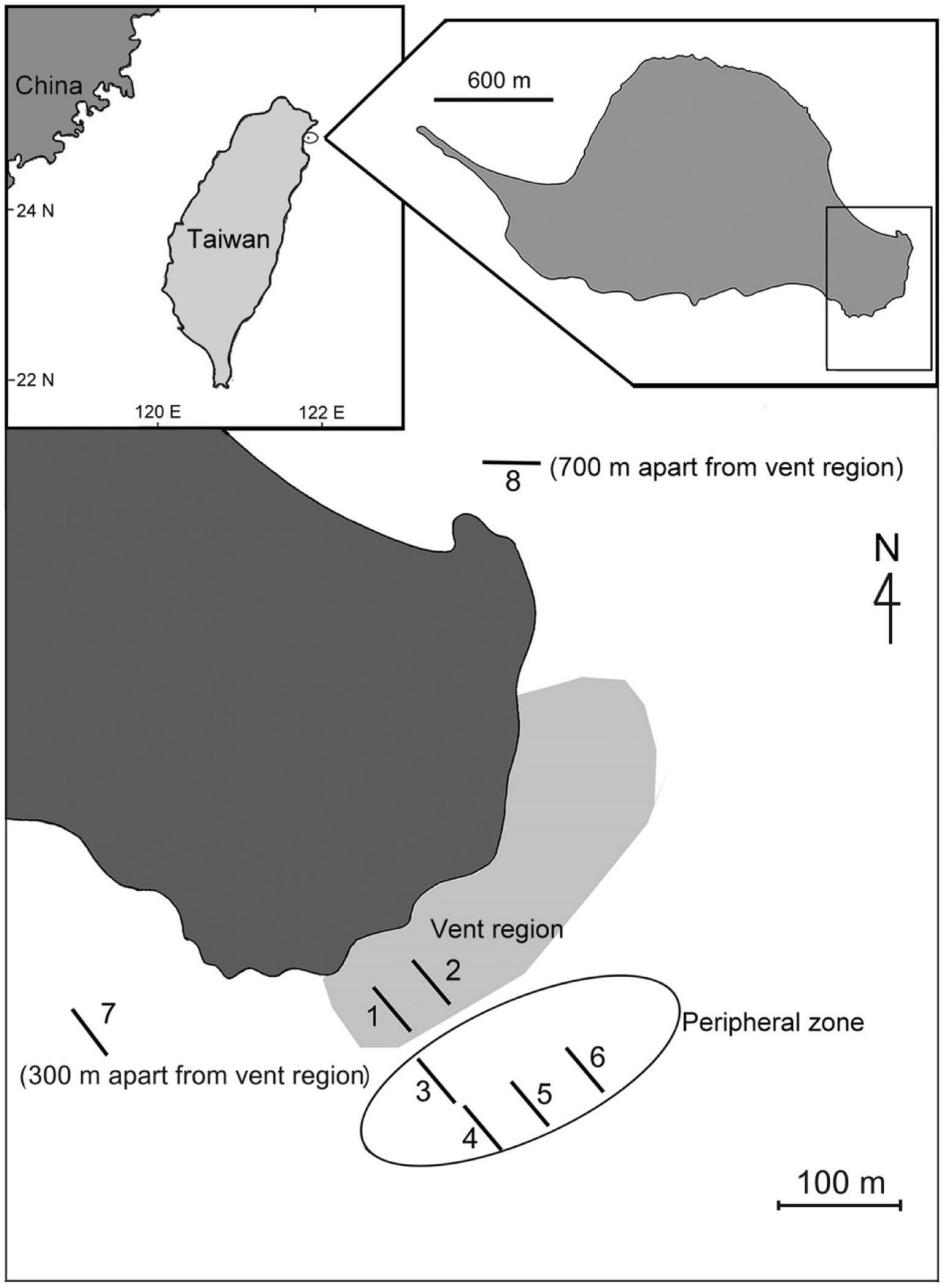
Kueishan Island and sampling locations of food sources and consumer groups for δ^13^C and δ^15^N analysis ( modified from Chan et al.^12^).

Kueishan Island is located in northeastern Taiwan (Fig. 1) and well known for having high densities (> 30 vents/km^2^) of both shallow- and deep-water hydrothermal vents ranging from 10 to > 300 m in depth^12,19,20^. Two types of shallow-water vents are found near the island, which are characterized mainly by their different temperature ranges. The yellow vents are hot and acidic (78–116 °C and pH 1.52–6.32 at vent openings), whereas the white vents are relatively cooler but have similar acidity (30–65 °C and pH 1.84–6.96)^12^. Similar to the gas from fumaroles and hydrothermal vents elsewhere, the gaseous discharge from vents at Kueishan Island mainly comprises CO_2_ (90–99%) and H_2_S (0.8–8.4%), with a minor fraction of SO_2_ (< 0.03%) and HCl (< 50 ppm)^12,19^. The heat and the inorganic and organic compounds released from these vents create a strong gradient in the biogeochemical environment (e.g., decreasing water temperature and increasing pH and dissolved oxygen), consequently leading to increases in species richness and biodiversity from vent mouths to peripheral regions^12,21–23^.

The vent crab *Xenograpsus testudinatus* Ng, Huang, and Ho, 2000 is the most abundant species concentrated around shallow-water vent openings near Kueishan Island^24^, and its density decreases with distance from the vents^12,20,24^. It is an omnivore or scavenger with a diet that may comprise sediment, zooplankton killed by vent effluents^20,24^, or organic matter (OM) from chemolithotrophic bacteria (i.e., Epsilonproteobacteria)^16,20,25,26^. Chang et al.^16^ suggested that chemosynthetic production is more crucial than photoautotrophs for *X. testudinatus*, benthic primary consumers (e.g., epibenthic crustaceans), and other omnivores near (i.e., ≤ 100 m) the vent mouths of Kueishan Island because the trophic support from vent-associated OM is greater than that from other particulate OM collected at surface water or sea bottom on the basis of stable isotopes. However, Chang et al.^16^ did not consider the possibility of the direct consumption of benthic algae by the consumers (e.g., small epibenthic crustaceans and the vent crab). In addition, Chang et al.^16^ did not consider the chemosynthetic bacteria that grow on free rocks or the surface of crabs as a potential food source. This might have resulted in an overestimation of the trophic reliance on chemosynthetic production. Wu et al.^27^ compared the isotopic niches of the distinctive vent and nonvent regions in Kueishan Island, which are approximately 3–14 km apart, and discovered a niche differentiation of benthic consumers between the two regions. However, although the greatest physicochemical changes in the water occur near the vents, that is, ≤ 1 km from their center, the spatial differences in the isotopic niche of the benthic consumers near the vents are unknown.

In the nonvent region, the density of *X. testudinatus* substantially decreases at 150 m from the vents near Kueishan Island, where sea anemones and some molluscan taxa are more abundant, with their abundance peaking at 150–300 m from the vents^12^. Gradual changes in species, functional feeding groups, and the diversity of benthic consumers have been observed from regions near the vents to those 700–1000 m from the vents: from vent crabs (omnivores or scavengers), sea anemones (suspension feeders), slipper limpets (suspension feeders; see Collin, 2019^28^) in the vent region to snails (omnivores or scavengers), corals (suspension feeders) and cowries (grazers) in farther areas^12^. Similar patterns are observed in shallow-water vents elsewhere^3,9^. However, some general ecological questions remain, such as the changing role of chemosynthetic and photosynthetic trophic pathways along the horizontal distance from the vents and whether consumers of the same trophic guild (e.g., omnivores or suspension feeders) inhabiting regions near and far from the vents differ in their resource use (e.g., OM from vent effluents vs. zooplankton), especially when the resources differ in quality (i.e., zooplankton have a higher food-quality). The role of algae in benthic communities and the inter- and intraspecific variation in the trophic support and isotopic niches of fauna across the spatial and environmental gradient at the shallow-water vents have received little attention. Studies on deep-water vents revealed that organic matter produced by chemoautotrophic bacteria together with phytodetritus are important food sources for macrofauna. Some meio-fauna including nemantodes rely on non-vent food sources^3,29–32^. There is a sphere of influence of vents and the vent fluids can strongly affect the trophic connections between vent and non-vent habitats^6^.

We analyzed the stable carbon and nitrogen isotopes (i.e., δ^13^C and δ^15^N) in food sources (benthic algae and their detritus, algal films, bacteria, and zooplankton) and the benthic fauna of multiple feeding groups and trophic levels collected from shallow-water vents near Kueishan Island. We quantified the diet composition and isotopic niches of the benthic consumers by using a Bayesian mixing model, MixSIAR^33^. We hypothesized that taxa inhabiting regions near the vents (e.g., *X. testudinatus*) would be more dependent on chemolithotrophic bacteria and vent OM discharge, whereas those living farther away (e.g., sea anemones, corals, and gastropods) would be more dependent on photoautotrophs and zooplankton from both pelagic and benthic habitats. We also hypothesized that there is intraspecific variation in trophic base and isotopic niche of consumers along the gradient from vent to non-vent regions. For instance, conspecific suspension feeders living near the vents use multiple resources (i.e., a relatively equal use vent OM, chemolithotrophic bacteria, algae, and zooplankton) for biomass production, whereas those in distant regions have a more specialized diet (i.e., relying on only one or a few resources) because of the limited availability of vent OM and chemolithotrophic bacteria.

## Results

We classified the food sources into five groups: bacteria, algal films, algae + POM (i.e., macroalgae and deposited particulate OM), *Bornetella* (i.e., the green spherical algae *Bornetella sphaerica*), and zooplankton (see “Methods” section and Table 1 for statistical significance of stable isotopic variations among food sources within each group). The bacteria were the most ^13^C depleted, whereas the algal films were the most ^13^C enriched (Table 1). In addition, the algal films were the most ^15^N depleted, and the zooplankton had the highest ^15^N values (Table 1). The five food source groups significantly differed in both δ^13^C (Kruskal–Wallis test: H = 25.656, degree of freedom (df) = 4, p < 0.001) and δ^15^N levels (Kruskal–Wallis test: H = 25.866, df = 4, p < 0.001).

**Table 1:** δ^13^C and δ^15^N values of the five basal resource groups and their original sources collected from the Kueishan Island shallow-water vents. n, number of samples.

Group	Mean δ^13^C ± SD	Mean δ^15^N ± SD	Source	*n*	Average δ^13^C ± sd	Average δ^15^N ± sd
Bacteria (*n* = 12)	− 22.2 ± 0.7	5.8 ± 2.5	Bacteria from vent water	6	− 21.9 ± 0.9	6.5 ± 3.4
			Bacteria of vent crabs carapaces	6	− 22.5 ± 0.3	5.1 ± 0.9
Algae + DOM (*n* = 84)	− 19.9 ± 2.5	5.2 ± 1.6	*Cladophora dotyana* (Green algae)	26	− 19.2 ± 1.7	5.5 ± 0.9
			Green turf algae	15	− 22.4 ± 3.1	5.6 ± 1.6
			Deposited particulate organic matters (POM)	43	− 19.4 ± 2.2	4.9 ± 1.9
Zooplankton (*n* = 13)	− 19.6 ± 1.3	7.2 ± 1.0	Benthic plankton	4	− 18.9 ± 0.9	6.9 ± 1.0
			Surface plankton	9	− 19.9 ± 1.4	7.3 ± 1.0
*Bornetella sphaerica* (*n* = 2)	− 15.9 ± 0.1	2.2 ± 0.9	*Bornetella sphaerica*	2	− 15.9 ± 0.1	2.2 ± 0.9
Algal film (*n* = 1)	− 10.2	− 0.5	Algal film	1	− 10.2	− 0.5

Statistical analysis:

Bacteria from vent water and vent crab carapaces: (t-tests: [δ^13^C] t_0.05(2),9_ = 0.84, p = 0.44; [δ^15^N] t_0.05(2),9_ = 0.46, p = 0.67).

Green algae, Green turf algae and POM: (One way ANOVA [δ^13^C]: F_2,81_ = 11.05; p < 0.05, SNK test: Green algae = POM > Green turf algae; [δ^15^N], F_2,81_ = 1.74; p > 0.05).

Surface and Benthic zooplankton: (t-tests: [δ^13^C] t_0.05(2),11_ = -1.33, p = 0.22; [δ^15^N] t_0.05(2),11_ = 0.64, p = 0.53).

Significant differences in δ^13^C and δ^15^N were observed between the consumer taxa (Table 2). The δ^13^C values of the nonzooxanthellae corals *Tubastraea* spp. (Dendrophylliidae), sea anemones *Anthopleura* sp. (Actiniidae), grazing cowries (Cypraeidae), and slipper limpets *Bostrycapulus aculeatus* (Gmelin, 1791) (Calyptraeidae) were similar but significantly higher than those of the vent crabs *X. testudinatus* and neogastropod snails (pooled *Anachis misera* (Sowerby, 1844) (Columbellidae) and *Ergalatax contracta* (Reeve, 1846) (Muricidae); Table 2). Sea anemones and neogastropods were significantly more ^15^N-enriched than vent crabs, corals, and cowries (Table 2), whereas vent crabs, corals, and cowries did not differ in δ^15^N. Slipper limpets were the most ^15^N-depleted among the consumers (Table 2).

**Table 2:** δ^13^C and δ^15^N values (mean ± SD) of the benthic consumers collected from the Kueishan Island shallow-water vents. *n*, number of samples.

Station		Vent region	Peripheral zone (150–300 m from vent center)				300–700 m from vent center	
		1	3	4	5	6	7	8
**Suspension feeders**								
Sea anemone (*Anthopleura* sp.)	*n*	3	5	8	17	16	29	9
δ^13^C		− 19.7 ± 0.48	− 19.5 ± 0.07	− 19.8 ± 0.5	− 19 ± 0.43	− 18.9 ± 0.45	− 18.6 ± 0.91	− 17.3 ± 0.81
δ^15^N		8.2 ± 0.21	8.9 ± 0.16	9.4 ± 0.46	9.5 ± 0.33	9.4 ± 0.44	9.7 ± 0.4	9.8 ± 0.43
Slipper limpet *Bostrycapulus aculeatus*	*n*		5	2	2	9		2
δ^13^C			− 17.8 ± 0.52	− 18.1 ± 0.37	− 18.7 ± 0.59	− 18.4 ± 0.44		− 18.5 ± 0.09
δ^15^N			2.2 ± 0.72	4.5 ± 0.52	5.9 ± 1.17	4.8 ± 0.36		5.8 ± 1.94
Coral *Tubastrea* spp.	*n*				1	7	26	37
δ^13^C					− 21.8	− 19.2 ± 1.47	− 19.6 ± 1.08	− 20 ± 0.75
δ^15^N					8.6	8.3 ± 0.57	8.3 ± 0.59	8.4 ± 0.64
**Grazers**								
Cowrie	*n*					10	6	
δ^13^C						− 19.1 ± 1.24	− 17.8 ± 1.27	
δ^15^N						8.1 ± 1.02	8.8 ± 0.34	
**Predators/scavengers**								
Vent crab (*Xenograpsus testudinatus*)	*n*	6		40	156	10		
δ^13^C		− 16.9 ± 0.77		− 17.2 ± 1.34	− 16.6 ± 1.03	− 16.9 ± 0.66		
δ^15^N		8.1 ± 0.94		7.5 ± 1.01	7.2 ± 1.43	8.3 ± 1.17		
Neograstropods (*Anachis* & *Ergalatax* spp.)	*n*		5	9	13	21		
δ^13^C			− 17.4 ± 1.06	− 17 ± 0.78	− 17.3 ± 0.93	− 16.6 ± 0.6		
δ^15^N			8 ± 0.37	9.1 ± 0.67	8.6 ± 0.6	9.5 ± 0.94		

**Variation among consumers (stations pooled)** Kruskal Wallis tests: (δ^13^C: H = 236.47, df = 4, P < 0.001; δ^15^N: H = 25.866, df = 4, P < 0.001).

Dunn’s test: δ^13^C: sea anemone = cowrie = slipper limpet > vent crabs = neogastropods; δ^15^N: sea anemone = neogastropds > vent crab = corals = cowries > slipper limpet.

**Variation among stations within each consumer.**

vent crabs: Kruskal–Wallis test: H = 7.628, df = 3, p > 0.05.

sea anemones: ANOVA: F_6,80_ = 13.069, p > 0.05;

neogastropods: ANOVA: δ^13^C: F_3,44_ = 2.66, p < 0.05; SNK tests: 8 > 5 = 6 = 7 > 1 = 3 = 4; δ^15^N: F_3,44_ = 8.576, p < 0.05; SNK tests: 8 > 4 = 5 = 6 = 7 > 1 = 3.

slipper limpets: ANOVA: δ^13^C: F_2,17_ = 3.312, P > 0.05; δ^15^N: F_2,17_ = 22.08, p < 0.05; SNK tests: 4 = 5 = 6 = 8 > 3.

corals: ANOVA: δ^13^C: F_2,67_ = 4.5, p > 0.05; δ^15^N: F_2,67_ = 0.172, p > 0.05.

cowries: t-tests: δ^13^C: t =  − 2.04, df = 14, p > 0.05; δ^15^N: t = 1.535, df = 14, p > 0.05.

**Figure 2:**
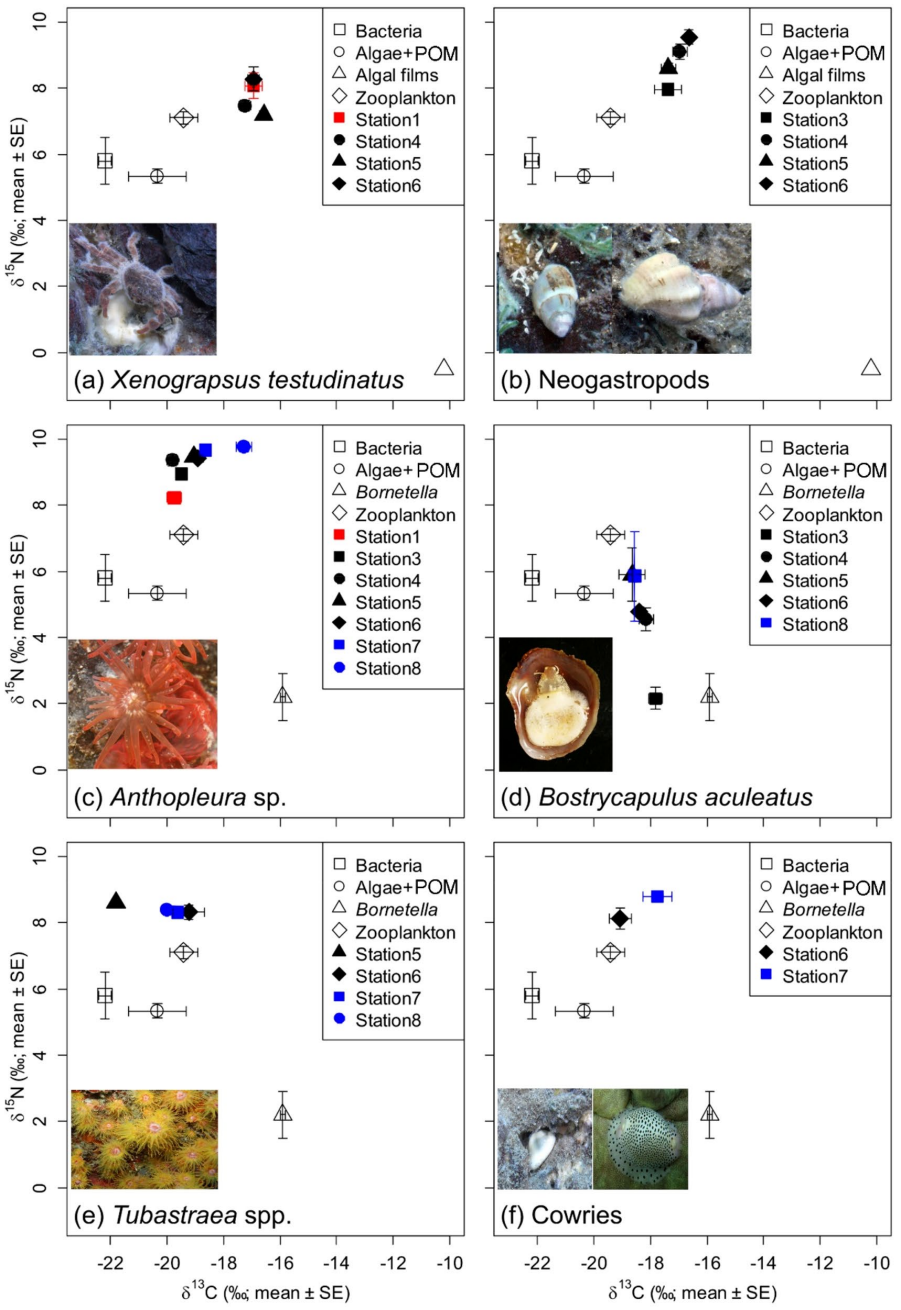
Biplots of δ^13^C and δ^15^N (mean ± SD) for (**a**) vent crab *Xenograpsus testudinatus*, (**b**) neogastropods, (**c**) *Anthopluera* sp., (**d**) *Bostrycapulus aculeatus*, (**e**) *Tubastraea* spp., and (**f**) cowries (solid symbols) from different sampling stations and their potential food sources (open symbols). Isotopic values of food sources do not include trophic fractionations.

**Figure 3:**
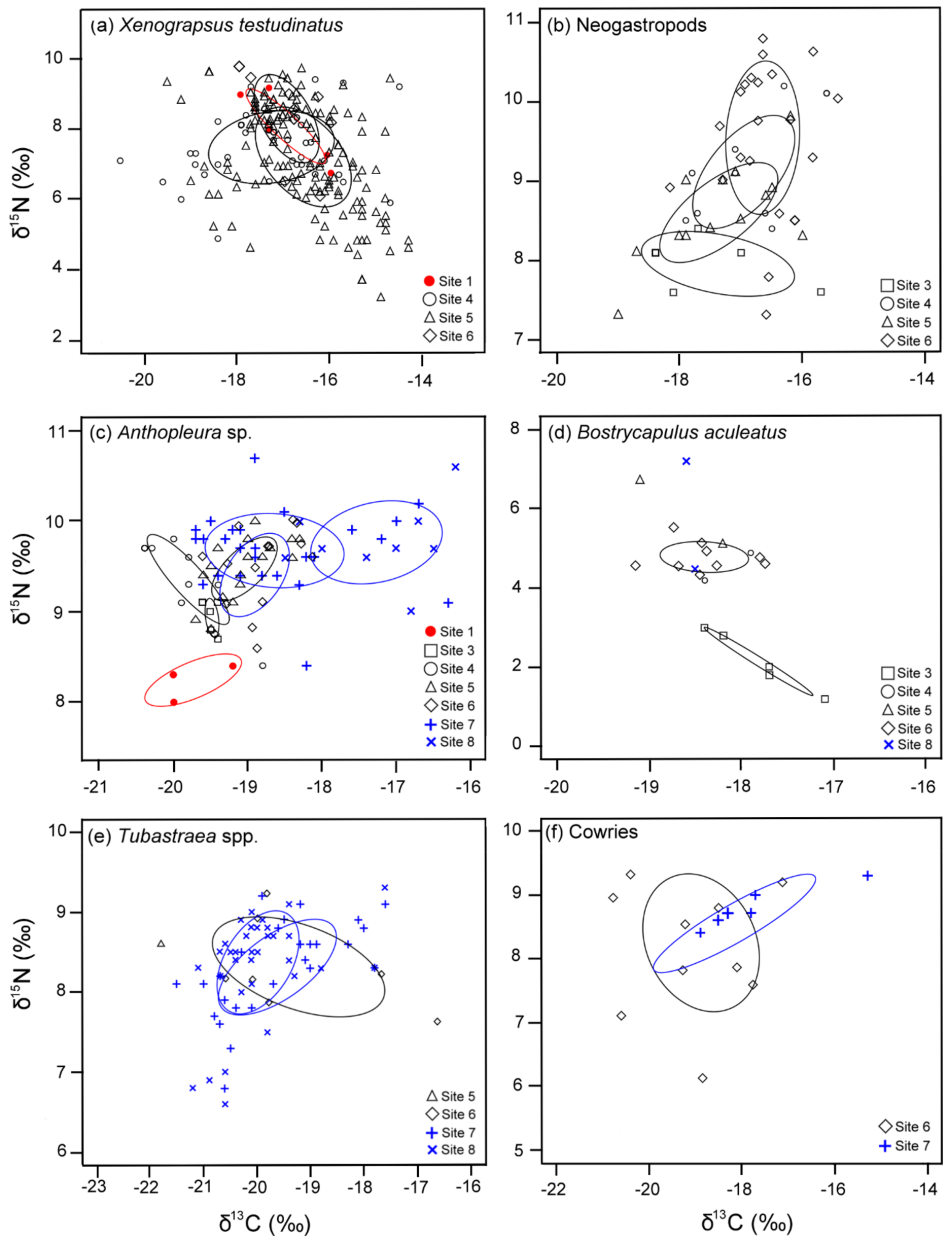
The Bayesian standard ellipses area (SEAc) based on δ^13^C and δ^15^N data for (**a**) vent crab *X. testudinatus*, (**b**) neogastropods, (**c**) *Anthopluera* sp., (**d**) *B. aculeatus*, (**e**) *Tubastraea* spp., and (**f**) cowries at each station. SEAc was calculated only if sample size was > 2.

**Figure 4:**
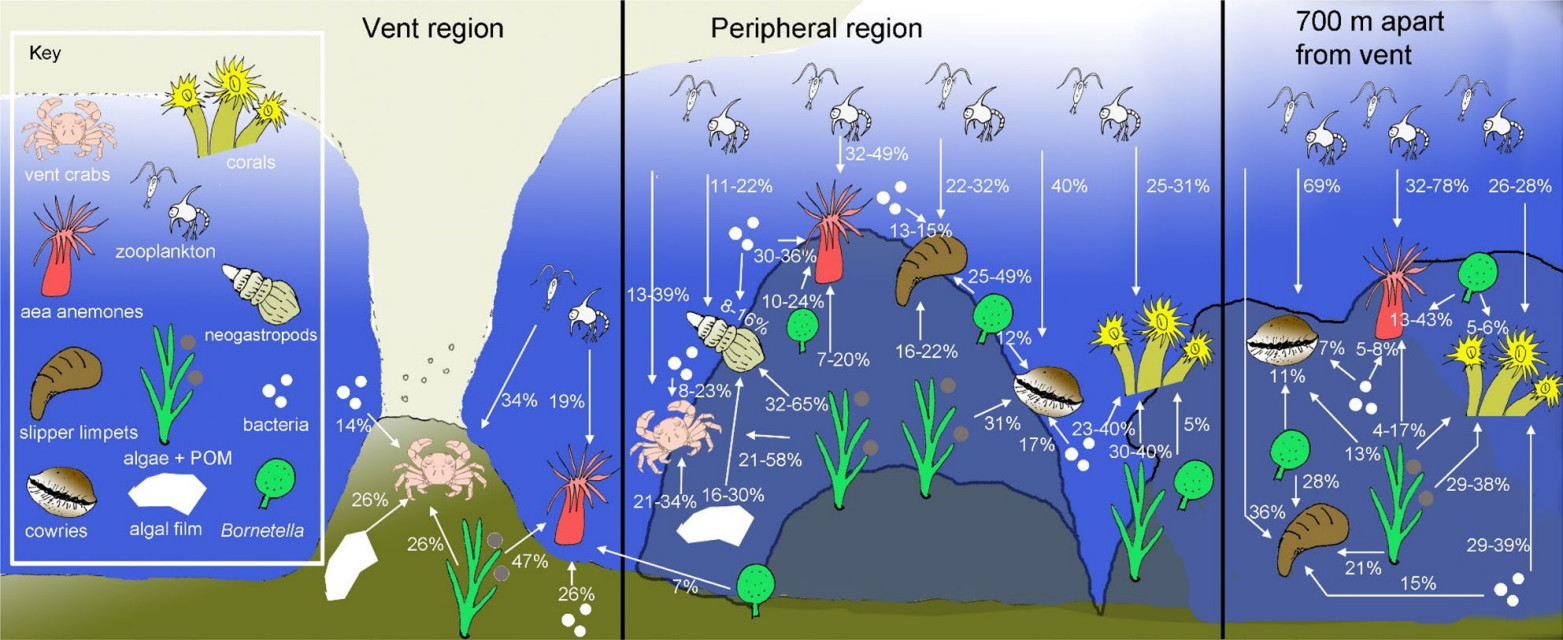
Schematic summary diagram to show the relative contributions (%) of individual food sources to the consumers (see Table 3) near the vent mouths, in the peripheral region, and at 300–700 m apart from the vent mouths.

Vent crabs were present at both vent mouths and the peripheral regions, that is, 150–300 m from the vent mouths (Table 2; Fig. 2a), and exhibited a wide range in δ^13^C (− 20.5 to − 14.3‰) and δ^15^N (3.2 to 9.8‰). Gradual transitions in their isotopic niche were observed across the regions, as indicated by the corrected Bayesian standard ellipse area (SEAc) (Table 2; Fig. 3a). The δ^13^C and δ^15^N of the vent crabs did not differ across regions (Table 2). At Station 1, zooplankton were the main food source for vent crabs, and the dietary contribution of algae + POM was similar to that of algal films (Table 3). The dietary contribution of algae + POM and algal films was consistently greater than 0.2 (or 20%) in all stations where the crabs were found. The vent crabs generally had the lowest diet dependence on bacteria, except those in Station 5. Their trophic reliance on zooplankton was high (> 34%) in Stations 1 and 6 but lower (≤ 18%) in Stations 4 and 5 (Table 3; Fig. 4).

**Table 3:** Mean trophic contributions of basal resources to the benthic consumers collected from the Kueishan Island shallow-water vents, based on the Bayesian mixing models using MixSIAR. Contributions are on a scale from 0 (0%) to 1 (100%).

Station		Vent region	Peripheral zone (150–300 m from vent center)				300–700 m from vent center	
		1	3	4	5	6	7	8
**Suspension feeders**								
*Anthopleura* sp. (sea anemones)	*n*	3	5	8	17	16	29	9
Algae + POM		0.47	0.20	0.05	0.07	0.10	0.04	0.17
Bacteria		0.26	0.30	0.36	0.31	0.34	0.05	0.08
*Bornetella sphaerica*		0.07	0.11	0.10	0.21	0.24	0.13	0.43
Zooplankton		0.19	0.39	0.49	0.41	0.32	0.78	0.32
*Bostrycapulus aculeatus* (slipper limpets)	*n*		5	2	2	9		2
Algae + POM			0.16	0.20	0.23	0.22		0.21
Bacteria			0.13	0.14	0.15	0.15		0.15
*Bornetella sphaerica*			0.49	0.35	0.25	0.31		0.28
Zooplankton			0.22	0.31	0.37	0.32		0.36
*Tubastrea* spp. (corals)	*n*				1	7	26	37
Algae + POM					0.30	0.40	0.38	0.29
Bacteria					0.40	0.23	0.29	0.39
*Bornetella sphaerica*					0.05	0.05	0.05	0.06
Zooplankton					0.25	0.31	0.28	0.26
**Grazers**								
Cowrie	*n*					10	6	
Algae + POM						0.31	0.13	
Bacteria						0.17	0.07	
*Bornetella sphaerica*						0.12	0.11	
Zooplankton						0.40	0.69	
**Predators/scavengers**								
*Xenograpsus testudinatus* (vent crabs)	*n*	6		40	156	10		
Algae + POM		0.26		0.58	0.25	0.21		
Algal films		0.26		0.21	0.34	0.26		
Bacteria		0.14		0.08	0.23	0.14		
Zooplankton		0.34		0.13	0.18	0.39		
*Anachis* & *Ergalatax* spp. (predatory gastropods)	*n*		5	9	13	21		
Algae + POM			0.65	0.45	0.56	0.32		
Algal films			0.16	0.25	0.20	0.30		
Bacteria			0.08	0.12	0.10	0.16		
Zooplankton			0.11	0.18	0.14	0.22

The neogastropod snails were present only in the peripheral regions (Table 2; Fig. 3b). They were more ^15^N-enriched but had similar δ^13^C values to those of the vent crabs (Table 2; Fig. 2a,b). The isotopic niche of the snails appears changed progressively from Station 3 to Station 6 (Fig. 3b) but there were no significance differences in their δ^13^C and δ^15^N among stations (Table 2). The neogastropods exhibited the strongest diet reliance on algae + POM (32–65%) at these stations (Table 3; Fig. 4). The dietary contributions of algal films and zooplankton to the neogastropods were greater than that of bacteria at each station (Table 3; Fig. 4).

Sea anemones were the only fauna found in all three regions. Its δ^13^C and δ^15^N values were lower in Station 1 than in the other sites (Table 2). They gradually became more ^13^C- and ^15^N-enriched from Station 1 to Station 8 (Table 2; Fig. 3c). The δ^13^C of sea anemones was lowest at Stations 1, 3, and 4, intermediate at Stations 5–7, and highest at Station 8 (Table 2). Similarly, the δ^15^N of sea anemones was lowest at Stations 1 and 3, intermediate at Stations 4–7, and highest at Station 8 (Table 2).

The sea anemones’ reliance on algae + POM was highest at Station 1 but gradually decreased in stations farther from the vents (Table 3). The dietary contribution of bacteria to sea anemones was consistently high in the vent mouth region and the peripheral zone (26–36%; Table 3; Fig. 4) but substantially decreased at Stations 7 and 8 (< 8%; Table 3; Fig. 4). By contrast, the sea anemones’ dietary dependence on *Bornetella* was low at Stations 1, 3, and 4 (< 11%; Table 3, Fig. 4) and increased at Stations 5–8 (13–43%; Table 3; Fig. 4). The trophic support from zooplankton for sea anemones was generally high (> 19%) and peaked at Station 7 (78%).

Slipper limpet was found in the peripheral and distant areas but not near the vent openings (Table 2; see also Chan et al.^12^). Overall, slipper limpets were more ^15^N-depleted than sea anemones (Table 2; Fig. 2c,d). Slipper limpets had significantly higher δ^15^N values at stations farther from the vent mouths (Table 3). The δ^13^C levels of the slipper limpets did not differ among stations (Table 3). The isotopic niche of the slipper limpets differed between Station 3 and the other sites (Fig. 3d), but the MixSIAR results indicated that *Bornetalla* and zooplankton were consistently the most important food sources (≥ 62% combined contribution) for slipper limpets at all sites, with their total dietary contribution peaking at Station 3 (71%) (Table 3; Fig. 4). The slipper limpets’ trophic reliance on algae + POM (16–23%) and bacteria (13–15%; Table 3; Fig. 4) was low.

Unlike the sea anemones and slipper limpets, corals were only present at stations distant from the vent mouths (Table 2), likely because of the physiochemical constraints caused by vent effluents^12^. Corals from Stations 5–8 overlapped in the isotopic space (Fig. 3e). No significant differences in δ^13^C and δ^15^N of the corals in Stations 5–8 were observed (Table 2). However, the Bayesian standard ellipses area (SEAc) results indicated that the isotopic niche of the corals was narrower at Stations 7–8 than that at Station 6 (Fig. 3e). The corals had similar trophic dependence on algae + POM (29–40%), bacteria (23–40%), and zooplankton (25–31%) (Table 3). The trophic support from *Bornetella* was relatively low (< 6%).

Cowries were found only at Stations 6 and 7 (Table 2). In total, four genera were present: *Cypraea*, *Erronea*, *Monetaria*, and *Nereinid*. No differences in δ^13^C of the cowries were observed among stations. Their isotopic niche was narrower in Station 7 (Fig. 3f). However, the results from the MixSIAR models indicated that zooplankton (41–69% contribution) were consistently the main energy source for the cowries at both sites (Table 3). The trophic dependence of cowries on algae + POM was lower than that on zooplankton but was still considerable (31%; Table 3; Fig. 4) at Station 6. The dietary contribution of bacteria and *Bornetella* to cowries was generally low (≤ 17%).

The diet of the shallow-water vent fauna was mainly supported by algae + POM (contribution for vent crabs: 26%; sea anemones: 47%) or zooplankton (vent crabs: 34%; sea anemones: 19%) rather than by chemosynthetic bacteria (vent crabs: 14%; sea anemones: 26%).

## Discussion

We found that bacteria were not the major trophic support for consumers at the shallow-water vents overall, which did not support our first hypothesis. At the vent mouths, the dietary contribution of chemosynthetic production (i.e., chemolithotrophic bacteria) to vent crabs and sea anemones was 14–26%, lower than that of both algae + POM (26–47%) and zooplankton (19–34%). At the peripheral zone and the region 300–700 m away from the vent mouths, the trophic reliance of the slipper limpets and nonsuspension feeders (i.e., the vent crabs, neogastropods, and cowries) on bacteria was lower than their reliance on all other food sources. The dietary contribution of bacteria was higher for sea anemones and corals in these two regions but rarely exceeded that of algae + POM or zooplankton. Thus, the consumers in the vent regions consistently relied more on algal resources and zooplankton. The zooplankton were likely supported by phytoplankton because they had distinct isotopic signatures from bacteria, and no apparent input of terrestrial OM from nearby coastal ecosystems was observed^34,35^. Our results indicated that the food chains and consumer production at shallow-water vents were mainly driven by photoautotrophs or phytoplankton through zooplankton rather than by chemosynthetic bacteria, although chemosynthetic bacteria can provide supplemental energy for the vent consumers.

Our findings are in contrast with those of Chang et al.^16^, which indicated that chemosynthetic production is the trophic base in shallow-water vents. This is likely because Chang et al.^16^ did not consider benthic algae as a potential food source while quantifying the diet composition of small epibenthic crustaceans and vent crabs, which might have resulted in an overestimation of the contribution of vent OM (i.e., bacterial). When Chang et al.^16^ included benthic algae in the analysis of the food sources of neogastropods, which had highly similar isotopic signatures to those of their vent crab samples, they discovered a substantial trophic reliance of these neogastropods on algae (44 ± 6% mean ± standard deviation (SD) for *E. contractus* and 32 ± 6% for *A. misera*). However, we did not exclude the possibility of temporal variation in the trophic support of the vent food chains (samples in this study were collected in 2014 and in 2015 for Chang et al.^16^) because the study region is seismically active and occasional, small seismic activity may affect vent structure, the supply of OM^22^, and algal abundance and distribution.

Our results revealed that suspenson feeders have greater trophic flexibility among taxa when compared to the omnivores. Among the omnivores, both vent crabs and neogastropods exhibited a high trophic reliance on the benthic algal resources (i.e., algae + POM and algal films; total contribution = 47–80% and 62–81% for the vent crabs and the neogastropods, respectively) at Stations 4–6, where they coexisted. We observed a greater reliance of vent crabs on algae + POM and algal films in the outer peripheral zone (i.e., Stations 5 and 6) (Table 2; Fig. 4). This indicated that, in the peripheral region, vent crabs also randomly preyed on fauna (or scavenged their carcasses), such as the slipper limpets or sea anemones, which had high trophic reliance on algae + POM and *Bornetella*. Slipper limpets and sea anemones were perhaps the main prey that transferred algal energy and nutrients from distant regions to the vent crabs in the peripheral zone. In addition, some neogastropod individuals were considerably more ^13^C- and ^15^N-enriched than most of the food sources (i.e., except algal films; Fig. 2b). This was possibly the result of cannibalism^36–38^ or the scavenging of carcasses from higher trophic levels (e.g., fish), whose tissue was enriched in ^13^C and ^15^N, because a missing basal food source with high ^13^C and ^15^N values is unlikely. Although the overall algal support for the vent crabs and the neogastropods was similar, the algal energy may have been channeled to these omnivores through different trophic pathways. Vent crabs can move among stations in vent and peripheral regions and can transfer trophic energy between these two regions.

In contrasts to the omnivores, the benthic suspension feeders exhibited larger differences in diet and isotopic niche among taxa. Sea anemone and corals relied mainly on energy from algae + POM, zooplankton, or bacteria, whereas the slipper limpets *B. aculeatus* consistently depended more on *Bornetella* and zooplankton. Although sea anemones can move using its basal disc when the environment is not suitable, their movement range is often within metres^39^. Chang et al.^16^ analyzed the diet compositions of sea anemones and corals with only two food sources (i.e., epibenthic crustaceans and zooplankton) and observed an even higher contribution from zooplankton for these suspension feeders compared with our estimates. Sea anemones and coral were also more ^15^N-enriched and thus had higher trophic positions than slipper limpets in our study. Coexisting suspension feeders were expected to have a homogeneous energy source^9^, but our study provided evidence that they could differ in feeding or assimilation selectivity and efficiency for ingested food particles. Dando^9^ proposed inferring dietary differences between coexisting suspension feeders on the basis of their morphology and feeding behavior. Sea anemones and corals are sessile but have tentacles to facilitate capturing zooplankton, which are a major source of their energy and essential nutrients (e.g., proteins, fatty acids)^40,41^. *Tubastraea* corals do not have symbiotic zooxanthellae and are exclusively suspension feeders^42^, unlike other Scleractinia corals that have photosynthetic symbionts for trophic support^43,44^. Slipper limpets do not have specialized structures for capturing prey, but along with suspension feeding, they might have foraged over a wider area closer to the seafloor and ingested more algae and POM. The differences among the diets of suspension feeders could have reduced the competition for resources, and the foraging behavior of slipper limpets likely increased the import of algal energy and nutrients from farther areas to support the vent food chains.

Our results support the hypothesis that conspecific consumers at shallow-water hydrothermal vents vary spatially in trophic base and isotopic niche corresponding to the availability of different food resources along the physicochemical gradient created by vent plumes. This intraspecific variation was observed in both suspension feeders and omnivores and was more apparent in sea anemones, slipper limpets, and neogastropods. For instance, the vent mouth region was not optimal for zooplankton survival and growth because of the high temperature and low pH and dissolved oxygen level^12^, resulting in lower zooplankton density and their trophic support for sea anemones. Therefore, the sea anemones derived more energy from algae + POM and bacteria near the vent openings. However, zooplankton became the major diet (32–78% contribution) of sea anemones when the environment was more favorable for zooplankton in peripheral and distant regions, where the reliance of sea anemones on algae + POM decreased. Similarly, the trophic reliance of slipper limpets on *Bornetella* generally decreased (from 49% to 28%), whereas that on zooplankton increased (from 22% to 36%) in stations farther from the vent mouth region. The neogastropods were also less dependent on algae + POM (a decrease from 65% to 32%) between Stations 3 and 6, but their reliance on all other food sources increased. The contribution of different diet sources to the neogastropods was more similar at Station 6 than at Station 3, which might be due to the increased predation of the neogastropods on a wider range of animals (e.g., slipper limpets and cowries) that were more abundant in the outer peripheral region^12^. Despite the intraspecific variation in the trophic base and isotopic niche of certain animals, the overall pattern was a general increase in zooplankton support for the food chains farther from the vent mouths. Similar pattern was also reported in the deep-sea vent in the Myokin Knoll in the West Pacific^31^. Harpaticoid copepods have their δ^13^C changed from −17‰ in the vent chimney to −28‰ at base of vent and then to −22‰ at non-vent region, suggesting that the harpaticod copepods obtain carbon from chemosynthetic bacteria and phytodetritus in the vents, but reply mainly on phytodetritus outside vent region^4,31^.

Cowries were not observed near the vents, possibly because of the unfavorable physicochemical conditions and high predation pressure^12^. Intraspecific differences in the trophic support and isotopic niche of the cowries, therefore, were not apparent because of their limited distribution (i.e., Stations 6 and 7). Our results indicated that zooplankton constituted a substantial part of the cowry diet, contrasting the notion that cowries are mainly grazers. Zooplankton could have been killed by vent plumes and descended to the seafloor^22^, where cowries were able to consume them either deliberately or accidentally during grazing. Another cowry species, *Luria cinerea* (Gmelin, 1791), has been reported to graze and feed on reef sponges at Carrie Bow Cay in Belize^45^. However, cowries at Kueishan Island obtained a considerable amount of energy from benthic algae, reflecting their unselective grazing and assimilation strategies and can be considered as omnivorous grazers. The trophic contribution of chemolithotrophic bacteria to the cowries was limited. Other gastropod grazers (e.g., *Haliotis cracherodii* Leach, 1814 (Haliotidae) and *Lottia limatula* (Carpenter, 1864) (Lottiidae)) obtain substantial nutrition from the predominant benthic mats of sulfur-oxidizing bacteria (i.e., *Thiothrix* sp. (Thiotrichaceae)) at vent mouths^9,17^. The limited contribution of bacteria to cowries in our study could be related to the lower food quality and abundance of bacteria than that of zooplankton and the algal resources.

In conclusion, our results indicated interspecific and intraspecific differences in the trophic support and isotopic niche of multiple benthic consumers along the environmental gradient created by shallow-water vents. These differences may reflect reduced competition and optimized resource utilization among consumers. Despite the predominance of chemolithotrophic bacteria and the possible light limitation caused by the discharge of POM at vent mouths (i.e., Station 1)^12^, photosynthetic autotrophs (i.e., benthic algae + POM and algal films or *Bornetella*) are the main energy source for the consumers. Vent plumes can indirectly support the benthic food chains by killing or narcotizing zooplankton that eventually sink to the seafloor and become available for the benthic consumers, such as vent crabs^24^. Zooplankton could thus contribute to 34% of the crab biomass at vent openings. At a larger spatial scale, the overall trophic support from photosynthetic autotrophs (26–54%) and zooplankton (19–34%) is generally greater than that from chemosynthetic autotrophs (14–26%) for all vent consumers, regardless of the distance from the vents (Table 3). The vent-induced physicochemical gradient strongly structures not only the community composition^12^ but also the trophic ecology of the consumers. Such gradient and changes in community composition are also evident in shallow-water vents elsewhere^3,9^. Although trophic reliance on symbionts (e.g., endosymbiotic chemolithotrophic bacteria) underlies consumer production and biodiversity in deep-sea vents^3^, the strong reliance on photoautotrophs and the niche differentiation within and between consumer taxa can be common in shallow-water vent food webs worldwide.

## Methods

### Sampling sites and methods

Because the waters around Kueishan Island are outside protected areas, specific permission was not required for sampling. In addition, no endangered or protected species were collected in this study. Benthic fauna and potential food sources in the shallow-water vents were sampled at the same eight stations (mean depth, 10–18 m) as described by Chan et al.^12^ (Fig. 1). Stations 1 and 2 (Fig. 1) were located in the vent areas where both yellow and white vents were present, and the sediment comprises sulfur sands with various sizes of sulfur balls formed by the accumulation of particulate sulfur from vent plumes. Stations 3 to 6 were peripheral regions located 150–300 m from the center of the vent, where Station 1 was located. These areas did not have any obvious vent mouths, but occasional gas bubbling was observed from the seabed at the boundary region of Station 1 and Station 3. Stations 7 and 8 were located 300–700 m from Station 1, where no vent or gas bubbling was observed.

The sampling was conducted by scuba dives at 10–20 m depths (depending on seafloor topography) along a 50 m transect at each site from June to July 2014^12^. The six most abundant groups of consumers that were present in at least two stations were identified and collected. These consumers were vent crabs *X. testudinatus*; suspension feeders, namely sea anemone *Anthopleura* sp. (Actiniidae), slipper limpet *B. aculeatus* (Gmelin, 1791) (Calyptraeidae), and nonzooxanthellae corals *Tubastraea* spp. (Dendrophylliidae); grazing cowries (Cypraeidae); and omnivorous or scavenging neograstropod snails (pooled *A. misera* (Sowerby, 1844) (Columbellidae) and *E. contracta* (Reeve, 1846) (Muricidae)) (Table 1). At the vent mouth regions, the consumers (*X. testudinatus* and *Anthopleura* sp.) were collected mostly at Station 1 because Station 2 had lower faunal abundance.

A total of 11 potential food sources were collected from the sites (Table 1). Five benthic algal groups were hand-picked or scraped from the surface of rocks. The green algae *Cladophora dotyana* W. J. Gilbert, 1965 (Cladophoraceae) and mixed-species green turf algae were present from the vent mouths to distant regions^12^. These algae were sampled from vent and the adjacent regions. However, green spherical algae *B. sphaerica* (Zanardini) Solms-Laubach, 1892 (Dasycladaceae) was found only at Stations 7 and 8. A handheld plankton net (300 μm mesh, i.e., phytoplankton were excluded) and light traps (specifications follow those of Chan et al.^46^) were used to collect surface and benthic zooplankton, respectively. The surface zooplankton collected by the plankton net mainly comprised copepods (3 trawls at each of Stations 1, 5, 8). The benthic zooplankton collected by the light traps (two traps at Station 2, one trap at Station 4 and one trap at station 8) comprised fish larvae, shrimp and crab larvae, Cumacea, and mysids. From one-way ANOVA analysis, there were no significant differences in δ^13^C and δ^15^N signatures of surface zooplankton (δ^13^C: F_2,6_ = 4.02, p > 0.05; δ^15^N: F_2,6_ = 2.05, p > 0.05) among stations. Chemolithotrophic bacteria (mainly Epsilonproteobacteria^24^) were sampled by collecting plumes (10 L) from both white and yellow vent mouths directly at Station 1. Bacteria were also collected from 10 L of white surface water (0.5 m below surface) in Station 2. These plume and water samples were filtered through sterilized 0.2-μm filters in the laboratory, and the material retained on the filter was collected for analysis. Only cellulose acetate membrane has 0.2 μm pore size which can retain bacteria samples over filtration. We are aware that cellulose acetate membrane filter can potentially contribute carbon contamination on samples. To minimize the contamination, the membrane filter was rinsed by deionized water before filtration. After filtration, the material retained on the membrane was carefully washed off to aluminum discs using glass pipettes to avoid any physical scrubbing on the filter paper. All aluminum discs and glass pipettes were autoclaved before usage. White filamentous Epsilonproteobacteria on vent crab carapaces were sampled through scraping on six crabs^26^ and combined to attain minimum sample weight for analysis. We collected white algal films on the surface of rocks near the vent mouths through scraping on 20 collected vent rocks at Station 1 and combined them into a single sample to attain minimum sample weight for analysis. Deposited particulate OM (POM) was collected from sediment sampled at each station using cylindrical cores of 20 cm in length and 10 cm in diameter. The organic layer of sediment was resuspended in 0.45 μm filtered seawater, and the water was filtered through pre-combusted (600°C 5 h) GF/C filter (pore size: 0.45 μm). OM filtered through the GF/C filter was collected. All GF/C filter was rinsed in 1 N HCL to remove any potential carbonate contamination before usage. All animal and food source samples were maintained at −80 °C before processing for stable isotope analysis.

### Stable carbon and nitrogen analysis

The soft tissue from the consumers was dissected, that is, their shells and internal organs were removed to avoid variation due to different tissue types^47^. The tissue was acidified with 1 N HCL to remove inorganic carbon. The algae were rinsed with deionized water to remove OM and sediment. Algae and algal films were acidified with 1 N HCL. All potential food sources (including bacterial and algal films) and consumer tissue were freeze-dried (Labconco Lyph-Lock 4.5, USA) and then pulverized using a pestle and mortar. Approximately 1 mg of the biological samples was analyzed for stable carbon and nitrogen isotopes (δ^13^C and δ^15^N) with an isotope ratio mass spectrometer (Thermo Finnigan Delta Plus XL [Thermo Fisher Scientific Inc., USA]) at the Stable Isotope Ratio Facility for Environmental Research of the University of Utah, USA. The analytical standards were Vienna Pee Dee Belemnite and air for δ^13^C and δ^15^N, respectively. The δ^13^C and δ^15^N values (in ‰) were calculated using (*R*_sample_/*R*_standard_) − 1, where *R* = ^13^C/^12^C or ^15^N/^14^N. The analytical precision (SD) was 0.2‰ for both δ^13^C and δ^15^N.

### Food sources and their combination for analysis

The contributions of the individual food sources to the consumers were quantified using the isotopic data and MixSIAR^33^. Unlike other mixing models, such as IsoError and IsoSource, MixSIAR can simultaneously incorporate the isotopic variability associated with consumers (end members), potential diet sources, and trophic enrichments, and can process multiple sources^27^. MixSIAR also considers prior information within the model and generates potential dietary solutions as true probability (posterior) distributions. It is frequently used in food web studies, particularly for determining the relative support of food sources to consumers^48,49^. For a specific consumer in our study, only the food sources found in its habitat range were included in the MixSIAR model. For instance, *Bornetella* was absent at Stations 1–6 and thus excluded in the MixSIAR analysis for the vent crabs. We calculated the means ± SD of the isotopic signals for the individual algal groups, benthic and surface zooplankton, and deposited OM from the samples obtained at each site. In the mixing models, the number of potential food sources should be limited, and the food sources with similar isotopic signatures should be combined to prevent underestimation in the model output and improve the model inference. Thus, we combined several closely related food sources for the analysis as follows (Table 1):

1. Bacteria: all bacterial samples collected from the water in vent mouths, surface water, and the carapaces of the vent crabs were pooled because they did not differ in their δ^13^C and δ^15^N levels (Table 1). They were considered “bacteria” as a whole for all consumers.
2. Algal films: the δ^13^C and δ^15^N signatures of the white encrusting algal films on the surface of rocks in the vent region were distinct from those of the other food sources.
3. Algae + POM: averaged δ^13^C of algae and POM ranged from −19.2‰ to −25‰, δ^15^N of algae and POM ranged from 3.2‰ to 5.5‰. From ANOVA analysis, δ^13^C of green turf algae is significantly lower than that of POM and green algae. There was no significant difference in δ^15^N of green turf algae, green algae, and POM (Table 1). However, the green algae and green turf algae were pooled because they co-occurred in the same habitat. The δ^13^C and δ^15^N signatures of the deposited POM (mainly comprising macroalgal fragments and phytodetritus under microscopes) were close to this algal group, indicating that the POM comprised of phytodetritus. We subsequently combined all the algae and deposited POM as a single food source.
4. Zooplankton: the surface and benthic zooplankton were grouped because their isotopic signatures were similar (Table 1). Zooplankton can be killed by vent effluents and settle on the seafloor for benthic consumers to consume^18^.
5. *Bornetella*: a macrospherically shaped algae on the perimeter of the study area, considered as a food source only for animals whose habitat included the distant surroundings.

In the MixSIAR analysis for vent crabs and neogastropods, we used bacteria, algal films, algae + POM, and zooplankton as the potential food sources. Bacteria, algae + POM, *Bornetella*, and zooplankton were considered potential food sources for the sea anemones, slipper limpets, corals, and cowries. Algae + POM was included in the MixSIAR models for suspension feeders because these algae were observed near the suspension feeders, and their degraded fragments were present in the water column after decomposition^20^. As a result, each consumer group had four potential food sources for the MixSIAR analysis.

The mixing models were constructed using the MixSIAR graphical user interface version (version 3.1.7)^50^ in R version 3.5.1^51^. The trophic fractionations in δ^13^C and δ^15^N from food sources to primary consumers (i.e., suspension feeders and grazers) were 0.50 ± 1.31‰ (mean ± SD) and 2.20 ± 1.77‰, respectively^47,52^. The vent crabs and neogastropod snails were omnivorous according to the gut content analysis and their feeding behavior^20,52^. Thus, we assumed that they had a trophic level of 2.5, and the overall δ^13^C fractionation from the basal resources was 0.75 ± 1.86‰. McCutchan et al.^52^ reported that the δ^15^N enrichment from invertebrate prey to predators was 1.40 ± 0.87‰. Therefore we employed an overall δ^15^N fractionation of 2.90 ± 1.97‰ for these omnivores. We applied the default prior for MixSIAR, which assumes that all combinations of food sources are equally likely, with each source having a mean marginal contribution of 1/(number of food sources) to the consumers. Finally, Markov chain Monte Carlo (with short, normal, and long runs) was used to estimate the proportional dietary contribution (i.e., on a scale of 0–1, i.e., from 0 to 100%), and we determined whether the model converged using the Gelman–Rubun and Geweke diagnostics^53,54^.

We calculated the Bayesian standard ellipses area (SEAc) to quantify the isotopic niche size of the individual taxa at each station with a sample size of > 2. The SEAc is less sensitive to changes in sample size compared with the estimation of niche space estimation with convex hulls^55^. We expected that the SEAc of the consumers would shift in position or size on the bivariate (i.e., δ^15^N vs δ^13^C) isotopic space if the niches of the consumers changed along the spatial gradient from the vent mouths to the distant regions.

### Data analysis

The δ^13^C and δ^15^N signatures of the food sources and consumers were compared using ANOVA and post hoc SNK tests. We also used ANOVA and SNK tests to compare the stable isotope signals of each consumer taxon among the sites. For heteroscedastic data, nonparametric Kruskal–Wallis tests and post hoc Dunn’s tests were performed for the comparisons. Because cowries were only observed in Stations 6 and 7, we used Student’s t-test to compare their δ^13^C and δ^15^N values between sites.
